# Beta-Glucans of Cereals: Functional and Technological Properties

**DOI:** 10.3390/nu15092124

**Published:** 2023-04-28

**Authors:** Anna Lante, Elisa Canazza, Paolo Tessari

**Affiliations:** 1 Department of Agronomy, Food, Natural Resources, Animals and Environment (DAFNAE), University of Padova, Viale dell’Università, 16, 35020 Padova, Italy; elisa.canazza.3@studenti.unipd.it; 2 Department of Medicine (DIMED), University of Padova, 35128 Padova, Italy

**Keywords:** β-glucans, dietary fiber, diabetes, functional foods

## Abstract

β-glucans are a polymeric dietary fiber characterized by β-(1,3) and β-(1,4) glycosidic bonds between glucose monomers. They are often used as thickeners, stabilizers, and fat substitutes in foods. The functional and technological quality of β-glucans is attributed to their origin/source, molecular weight, and structural properties. In particular, physical treatments such as drying, cooking, freezing, and refrigeration influence their molecular, morphological, and rheological characteristics. In addition to their useful technical qualities, β-glucans are recognized for their numerous beneficial impacts on human health. For this reason, the European Food Safety Authority (EFSA) has provided a positive opinion on health claims such as cholesterol lowering and hypoglycemic properties relating to oats and barley β-glucans. This paper provides insight into the properties of β-glucans and different treatments affecting their characteristics and then reviews the latest research on β-glucans as a functional ingredient for people with type 2 diabetes mellitus (T2DM).

## 1. Introduction

Diabetes is a major global health problem, and an estimated 537 million adults (aged 20–79 years) were affected by diabetes in 2021, with an expected increase to 783 million by 2045 [1]. More than 95% of people with diabetes belong to the category of T2DM, which is widely considered the result of a combination of genetic predisposition, insulin resistance, excess body weight, and physical inactivity [2]. Even though T2DM can be preventable to some extent, the burden of T2DM patients has nevertheless increased in most countries and even in adolescents in recent years [1,3]. The quality of life for diabetes patients is substantially compromised by persistent hyperglycemia and the associated consequences, which also place a greater financial strain on the public health system. Therefore, urgent intervention is needed to reduce the rising incidence of T2DM.

A large number of studies have demonstrated an inverse correlation between the intake of dietary fiber and the likelihood of developing type 2 diabetes. In addition, some randomized control trials in people with the condition have demonstrated that dietary fiber intake can significantly reduce hyperglycemia and insulin resistance [4,5,6]. Based on solid evidence, the International Diabetes Federation (IDF) recommends a sufficient dietary fiber intake as the basic ingredient of a healthy diet and a component of nutritional therapy to combat T2DM [7]. Intestinal microorganisms, or intestinal microbiota, can use dietary fiber, a vast family of food components that includes a range of carbohydrates that are resistant to mammalian digestion and absorption. At first, dietary fibers were categorized based on their solubility, viscosity, and fermentability. For example, β-glucans, pectin, guar gum, psyllium, and konjac are common soluble fibers used in blood glucose regulation [6]. They can reduce the rate of nutrient absorption in the small intestine because they are typically very viscous and capable of forming gels. Additionally, they are extensively fermented by the gut microbiota in the colon, primarily in the large intestine, producing generally advantageous by-products such as short-chain fatty acids (SCFAs), gases (CO_2_, CH_4_, and H_2_), and lactic acid. Acetate, propionate, and butyrate are the primary SCFAs produced by the fermentation process, and they are important signaling molecules and energy substrates for the host [8]. SCFA favorably influence the levels of Peptide YY (PYY), Glucagon-Like Peptide 1 (GLP-1), cholecystokinin, and ghrelin, i.e., hormones that are efficient in appetite control [9]. Insoluble fibers, including cellulose, some hemicellulose, and lignin, are a group of less fermentable fibers and are generally used as fecal bulking agents. They can reduce the intestinal transit time and increase fecal mass, relieving symptoms of constipation and improving bowel function [10]. The majority of clinical studies to far have either examined the overall amount of fiber or the differences between soluble (SDF) and insoluble (IDF) dietary fibers. Instead of just looking at total fiber intake or, at best, its solubility, current research has rather emphasized the necessity of assessing the consumption and effect of individual dietary fibers in health and illness [11].

β-glucans are frequently found in grains, fungi, algae, yeast, and some bacteria. The glycosidic linkages in cereal β-glucans are a combination of beta-1,3 and beta-1,4 glycosidic linkages; hence, the name (1,3;1,4)-beta-glucan. β-glucans have a high water-binding capacity, which leads to their physicochemical properties such as solubility, viscosity, and gelation [12]. For this reason, β-glucans are often used as thickeners, stabilizers, and fat substitutes in foods. In addition to their technical qualities, they exhibit many health-promoting properties. Based on scientific evidence accumulated over the past few decades, both the FDA and EFSA have approved the use of health claims to lower cholesterol and control the glycemic response of β-glucans from barley and oats [13,14,15,16,17].

The origin, molecular weight, and structural properties of β-glucans all have a direct impact on their functional characteristics. A product’s extraction, purification, processing, and storage procedures all have an impact on a product’s molecular weight and structural/conformational characteristics [18].

The aim of the review is to summarize recent research activities on cereal β-glucans to provide a basis for the critical understanding of the potential and possible applications of this underutilized functional ingredient in various food matrices.

## 2. Overview of the Different Types of β-Glucans Present in Nature

### 2.1. Sources of β-Glucan and Main Chemical Characteristics

β-glucans are non-starchy polysaccharides naturally present in the cell walls of several organisms and higher cultures. Therefore, humans unintentionally consume β-glucans through their regular diet. The primary sources of food β-glucan for humans are cereals (especially oats and barley), fungi, algae, and yeast [19,20]. As summed up in Table 1, depending on the extraction source, β-glucans have a characteristic and distinguishable size, structure, arrangement, and type of bonds and branches.

### 2.2. β-Glucans from Cereals

Cereal β-glucans are linear homopolymers formed by D-glucose residues bound primarily through two or three consecutive glycosidic β-(1,4) bonds separated by a single glycosidic β-(1,3) link. Longer segments with glucose residues consecutively bound by β-(1,4) glycosidic linkages and with a degree of polymerization (DP) of 5–28 are less frequent. There is no evidence that two or more adjacent glycosidic β-(1,3) bonds occur in cereal β-glucan chains [21]. The molecular characteristics of β-glucans are generally derived from the analysis of oligomers obtained from the depolymerization of polymers with a specification (1 → 3, 1 → 4) -β-D-glucan hydrolase (lichenase) releasing 3-O-β-D-cellobiose-D-glucose (trisaccharide unit, DP3) and 3-O-β-D-cellotriose-D-glucose (tetrasaccharide unit, DP4) representing 90–95% of total oligosaccharides, while longer oligosaccharides (DP ≥ 5) represent only 5–10% of the total. In brief, the molar ratio is considered as the ratio of cellotriose to cellotetraose units (DP3/DP4) following the depolymerization of the β-glucan polymer chain [18]. β-glucans from different cereal species share the same general molecular structure but differ based on the DP3/DP4 ratio, in the ratio of β-(1,3) and β-(1,4) glycosidic bonds, and in the molecular dimensions [21]. Among cereals with the highest concentrations of β-glucans are oats and barley; lower concentrations are also present in wheat, rye, and rice kernels [18].

As reported in Table 2, the content and the quality of cereal β-glucans can vary substantially depending on the cultivar, variety, and place of growth. The health claims allowed by the EFSA and FDA only apply to β-glucans from oats (OBG) or barley (BBG). Therefore, the β-glucans in these two cereals received the main scientific attention among all β-glucans derived from cereals. The distribution of β-glucans in cereals is very uneven.

The highest contents of cereal β-glucans are in the outermost layers [18]. Depending on the tissue considered (pericarp, aleuronic layer, and endosperm), their molecular structure changes. Therefore, the β-glucans in the outer layers have a lower solubility and a higher molar ratio in DP3/DP4 than the β-glucans located in the endosperm. In this regard, the DP3/DP4 molar ratio is the ratio of DP3 trisaccharide units (3-O-β-D-cellobiose-D-glucose) to DP4 tetrasaccharide units (3-O-β-D-cellotriose-D-glucose) [22]. In fact, the β-glucans present in pearling by-products show a higher molar ratio in DP3/DP4 (~3.3–3.6) than those present in flours or fiber-enriched fractions (~2.8–3) [21]. As also demonstrated by the study [23], insoluble β-glucans were found in whole wheat flour and oat bran compared to refined flour.

## 3. Physico-Chemical Properties of Cereal β-Glucan

### 3.1. Molar Ratio of β-Glucans of Cereals

The molar ratio (DP3/DP4) directly affects the functional properties of the polysaccharide. Segments bound by glycosidic β-(1,4) bonds in the β-glucan structure are considered semi-flexible, while the presence of breaks in the glycosidic β-(1,3) bonds results in twisting of the chain. Consequently, oat β-glucans, with a higher ratio of glycosidic β-(1,4) bonds to glycosidic β-(1,3) bonds, demonstrated a greater flow resistance and consequently higher viscosity than barley β-glucans [24]. With regards to the molecular weight (MW) of the cereal β-glucans, it is changeable [25]. According to the EFSA, the MW of β-glucans present in barley and oats can vary approximately between 50 and 2000 kDa [14]. Environmental conditions are mostly responsible for these variations, although extraction, purification, depolymerization, and analytical methodologies used to quantify MW can also have an impact [26].

According to Regand et al. (2011), the factors influencing homo-polymer MW include the physical state of β-glucan in plant material, endogenous β-glucanase activity, processing conditions, and storage conditions [27]. De Arcangelis et al. (2019) provided evidence that β-glucans with a high molar ratio are more resistant to endogenous β-glucanases and this could be an important factor from a technological point-of-view [28].

### 3.2. Solubility of Cereal β-Glucans

Under controlled temperature and pressure conditions, the solubility of β-glucans refers to the highest concentration of the component that may be diluted in water to create homogenous solutions of the polymer [29]. Despite being categorized as soluble fibers, β-glucans’ molecular irregularity is reflected in their solubility in water. Although β-glucan’s molecular structure permits numerous interactions and connections between its chains and water molecules, a high degree of polymerization may reduce its solubility [9]. The solubility of β-glucans is one of the most important chemical-physical characteristics as it determines their extraction and possible applications. The solubility of β-glucans is strongly influenced by their intermolecular aggregation states and high polymerization [30]. Cellulose-like segments in cereal β-glucans could contribute to the stiffness of molecules in a solution; adjacent β-glucan chains containing segments with adjacent β-(1,4) glycosidic bonds may show a tendency to intermolecular aggregation, and consequently to lower solubility, through hydrogen bonds along these portions. The bonds β-(1,3) glycosides break the regularity of the sequence β-(1,4) and make the molecule more soluble and flexible [26].

The solubility/extractability in water (under comparable conditions of time, temperature, pH, and other extraction conditions) of oat β-glucans is generally higher than those of barley, which in turn is greater than those of wheat. Therefore, solubility appears to decrease as the DP3/DP4 ratio along the β-glucan chains increases. In addition, the coexistence of different biopolymers in the cell wall, their spatial organization and the interactions between the components of the wall will likely affect the mechanical strength and permeability, thereby altering the solubility of the compounds [21]. The limited depolymerization of the polysaccharide increases its solubility in water and the stability of the solution. However, depolymerization from a low molecular weight β-glucans (i.e., 130,000 Da) could lead to a reduction in solubility as reported in oat bran muffins by Tosh et al. (2013) [31]. Furthermore, microwave treatment or germination (24 h) before the extraction of β-glucans from barley grains improves the extraction capabilities, as well as the nutraceutical properties. Methodologies are currently being studied to perform controlled depolymerization by acid or enzymatic treatment to improve the solubilization of β-glucans in water [18]. There is plenty of evidence indicating that solubility has less of an effect on the healthy qualities of β-glucans than previously believed, contrary to the conventional wisdom that only the soluble portion can have health advantages [32]. According to recent research by Khorasaniha et al. (2023) [11], ranking of the physiological advantages provided by fibers based on solubility has serious flaws. The solubility of dietary fiber, which varies across individuals, can be affected by the different pH levels in the gastrointestinal system. Because the pH of the solution varies throughout different organ systems where the fibers would be placed, the measurement of soluble and insoluble fiber content using in vitro techniques may not be equal to the solubility of the fibers in an in vivo environment (e.g., intestine). Since dietary fiber does not exist in a single subtype in all foods consumed, it can be challenging to estimate its volume. Additionally, because dietary fiber does not exist in a single subtype, interactions between dietary fiber and host cells or gut microbes can be influenced by simultaneous interactions between a variety of fiber and other nutrient subtypes in foods. In addition, a number of environmental factors can affect the content and solubility of dietary fiber, regardless of the effects of physiological factors [11].

### 3.3. Viscosity of β-Glucans in Cereals

There is a direct relationship between the increase in viscosity and the molecular weight of β-glucans, which is reflected in pseudo-elasticity characteristics and viscoelastic properties [26]. The behavior of β-glucan solutions is similar to that of a Newtonian solution at low concentrations; but, at concentrations over 1%, the molecules organize into a viscous, pseudoplastic complex that is typical of β-glucans. The development of a high viscosity solution is important for both technological and physiological reasons. The distribution of MW and concentration in a solution, limited by the solubility of the β-glucans themselves, are the major determinants of the viscosity of solutions containing β-glucans. In addition, depending on cultivar and environmental factors, the viscosity of β-glucan preparations can vary widely [18]. Oat β-glucans acted like high viscosity β-glucans, in contrast to barley β-glucans, which were described as low viscosity β-glucans and exhibited Newtonian behavior. In a comparison of the solutions containing comparable concentrations of oat and barley β-glucans, the solutions containing oat β-glucans had a viscosity approximately 100 times higher than that of the barley β-glucan solutions [33,34]. The optimal gelling temperatures for β-glucans from oats and barley are around 37 °C and 57 °C, respectively, according to studies on the effect of temperature on the gelling characteristics of barley and oats. When β-glucans are subjected to oxidative treatments, they can gel at the same temperature but produce weaker gels. Enzymatic and oxidative processes can be used to modify and enhance the viscosity of β-glucan extracts as well as their other functional qualities, making them more suitable for usage in the food industry. Nevertheless, oxidation might result in full depolymerization, especially at high temperatures. Thus, to preserve the required functional qualities of preparations containing β-glucans, conditions must be carefully controlled [18]. There are conflicting scientific opinions on the actual importance of the MW with reference to the viscosity of β-glucan solutions. Some authors argue that MW is directly correlated with the apparent viscosity of a solution containing β-glucans [33,35,36,37]. This would explain the lower viscosity of extracts obtained using digestive enzymes, thus leading to partially degraded β-glucans. However, other researchers claim that viscosity depends on the product between MW and soluble β-glucan concentration (MW × c) and not just MW [22,38]. Consequently, a reduction in MW does not necessarily cause a decrease in viscosity and net physiological properties. On the other hand, the study by Rieder et al. (2019) on the incorporation of β-glucans into bakeries revealed that there is no significant correlation between viscosity and reduced glycemic response after digestion in vitro [39]. This aspect is of particular interest, since, for most industrial applications, the high viscosity of β-glucans makes handling and processing more difficult.

## 4. Functional Properties of Cereal β-Glucans

**Figure 1:**
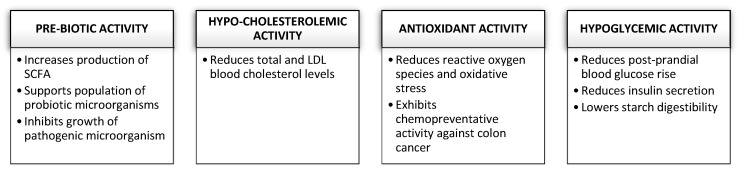
Health benefits associated with regular consumption of β-glucans from cereals adapted from [18].

β-glucans found in grains are recognized for their numerous beneficial impacts on the body, in addition to their useful technical qualities. The scientific community has focused its interest predominantly on β-glucans of barley and oats because of their ability to lower cholesterol and blood sugar. In addition to the two primary functional effects identified by the European Food Safety Authority (EFSA) and supported by health claims, a frequent consumption of cereal β-glucans has other significant advantages. Figure 1 summarizes the health advantages of a regular consumption of β-glucans from grains [18,40].

### 4.1. Prevention of the Onset of Diabetes by Cereal β-Glucans

#### 4.1.1. Epidemiology and Pathophysiology of Type 2 Diabetes Mellitus

As anticipated above, diabetes mellitus represents a major health problem throughout the world, and also with a variable penetration in different countries and income categories. Diabetes is responsible for 6.7 million deaths in 2021, or about 1 every 5 s. Just over half a billion people are living with diabetes worldwide, meaning that more than 10.5% of the world’s adult population now is affected by such condition [3,41]. Furthermore, over three out of four adults with diabetes live in low- and middle-income countries. The prevalence of diabetes in 2021 was estimated to be higher in urban areas (12.1%) than in rural areas (8.3%) and higher in high-income countries (11.1%) than in low-income countries (5.5%). In comparison to high-income (12.2%) and low-income (11.9%) nations, middle-income (21.1%) nations are predicted to see the biggest relative increase in diabetes prevalence between 2021 and 2045. Diabetes places a significant financial strain on health systems across the world. Healthcare costs associated with diabetes were predicted to reach USD 966 billion globally in 2021 and USD 1.054 billion by 2045.

From a pathophysiological standpoint, abnormally high blood glucose levels are brought on by a breakdown of the regulatory systems of insulin synthesis, release, and action. Beta cell dysfunction and apoptosis, insulin resistance, and defective metabolic mitochondrial activity are factors that contribute to type 2 diabetes mellitus and its etiology [42]. Due to hyperglycemia and the usually associated insulin resistance syndrome, also clustering in the “Metabolic Syndrome”, people with T2DM are at a significantly increased risk of both microvascular (retinopathy, nephropathy, and neuropathy) and macro-vascular (i.e., cardiovascular) complications [43].

Beyond insulin, however, the dysfunction of other hormones physiologically playing a role in glucose homeostasis, i.e., incretins and glucagon, is detectable in T2DM. Insulin secretion is also physiologically stimulated by two gastrointestinal hormones, i.e., the “incretins” GLP-1 (Glucagon-Like-Peptide 1) and GIP (Glucose-dependent insulinotropic polypeptide) [44]. Defects in the incretin secretion and/or the effect on T2DM contribute to a disturbed glucose homeostasis. The hypersecretion of glucagon, another pancreatic hormone produced by the alpha cells, increases hepatic glucose production and may impair the insulin peripheral effect, thus contributing to hyperglycemia [45]. Insulin resistance in adipocytes results in accelerated lipolysis and increased plasma levels of free fatty acids, which aggravate insulin resistance in the muscles and liver and contributes to beta cell failure. Furthermore, an increased renal glucose reabsorption by the sodium/glucose co-transporter 2 (SGLT2) and an increased threshold for urine glucose leakage, contribute to the maintenance of hyperglycemia. Resistance to the appetite-suppressing effects of insulin, leptin, GLP1, amylin, and PYY, as well as low brain dopamine and increased serotonin levels in the brain, contribute to weight gain, which exacerbates underlying resistance [43].

#### 4.1.2. Contribution of Diet β-Glucans to the Prevention of Type 2 Diabetes

The reduction in the postprandial glycemic response is among the most studied and documented properties of cereal β-glucans. A study conducted by Wolever et al. (2018) found that the meal addition with 1.6 g of oat β-glucans decreased the postprandial glycemic peak by 20% [46]. In this regard, EFSA has approved the health claim on the reduction in the postprandial glycemic response for foods containing at least 4 g of oat or barley β-glucans per 30 g of available carbohydrates [14]. A recent meta-analysis of β-glucan intake with cereals also concluded that β-glucans significantly reduce body weight and body mass index [47]. Overall, β-glucans play a critical role in reducing post-prandial glycemic and insulin responses. Due to their presence, the viscosity of the contents of the upper intestine increases, slowing the rate of starch digestion. Conversely, the three primary processes that contribute to the hypoglycemic impact of β-glucans are a limited access to digesting enzymes, a decreased luminal mixing, and a slower transport of glucose to the absorbent surface. Dong et al. (2011) found that the activity of three intestinal disaccharidases (*saccharidase, lactase,* and *maltase*) was inhibited by oat β-glucans in a dose-dependent manner both in vivo and in vitro, further contributing to a reduced glycemic response [48]. In view of the fact that digestible sugars are incorporated into this high viscosity gel, they will be less accessible to human digestive enzymes [27]. Therefore, it is plausible that viscosity is inversely related to the increase in blood glucose after meals [49]. However, although the link between the hypoglycemic effect and viscosity is evident, the role of the MW of β-glucans on the glycemic control is less clear. To date, there are still contradictory reports about the importance of molecular weight on such a physiological effect. Some researchers have found that both a high molecular weight and the viscosity of β-glucans are essential parameters for their nutraceutical properties, such as glycemic control [27]. Conversely, Rieder et al. (2019) concluded that MW does not significantly influence such a functional property attributed to cereal β-glucans [39].

In addition, a recent study in mice reported that the partial hydrolysis of barley β-glucans by endogenous β-glucanases (PHEB) did not impact their effects on the control of glycemic and LDL cholesterol [50]. Others [51] reported that even long-term daily consumption of 5 g of oat β-glucan/day has a beneficial effect on the metabolic control of subjects with T2DM, improving glycemic control, serum fatty acid profile, frequency of bowel evacuation, hormone regulation, and intestinal microbiota modification. Glycated hemoglobin A1c decreased by 0.68% after two weeks of therapy. These benefits may be connected to the numerous characteristics and actions of oat β-glucans, including their ability to gel at low concentrations (1%), at body-optimal temperatures, and under certain conditions [37]. In addition, a decrease in the expression of glucose transporters SGLT1 and GLUT2 and the inhibition of the enzyme α-glycosidase has been described [48]. While these latter properties regulate the absorption of carbohydrates, another effect could be that of an increase in GLP-1. This incretin also stimulates insulin release [52] through the generation of SCFAs (i.e., metabolic products of β-glucan fermentation by the intestinal microbiota), thereby stimulating L-, GLP-1 production [53]. The fermentation of β-glucans in the intestine and the consequent release of SCFA, also stimulated the Free Fatty Acid Receptor 2 (FFAR2), together with the activity and number of endocrine L-cells in the colon, thus increasing the basal peptide YY (PPY) release and the sense of satiety [54]. According to a study by Pino, Mujica, and Arredondo (2021), oat β-glucan may modulate intestinal bacterial populations in different ways. It significantly reduces butyrate-producing bacteria, primarily those belonging to the *Phylum Firmicutes*, and increases those belonging to the *Phylum Verrucomicrobia*, including *Akkermansia muciniphila*. The latter controls intestinal permeability while preserving the mucous layer, and it has been linked to better glycemic management, decreased insulin resistance, and lowered plasma cholesterol [55]. The decrease in *Phylum of Firmicutes* could be mediated by factors such as diet and medications; in T2DM subjects who undergo a modification of their gut microbiota, dietary fiber could reduce this overgrowth [51].

## 5. Cereal β-Glucans: Applications in Food Matrices

### 5.1. Application of Cereal β-Glucans in Food

The application of cereals β-glucans is desirable for various food products for their expected great advantages both in terms of technology and health. For technological purposes, β-glucans are mainly used as thickening agents, fat substitutes in light products, foam stabilizers, and emulsifiers. However, extraction, handling, and upstream processing treatments of β-glucans raise a number of practical challenges, particularly as a health ingredient, and their use is still limited [18]. Based on the rheological evaluation of hulled barley β-glucans, they can be incorporated into beverages and other liquid products as a thickener and as a source of dietary fiber [56]. However, the incorporation of β-glucans into foods is limited due to their high viscosity and the difficulties of industrial handling. This limitation is particularly evident when they are applied at sufficient concentrations to carry out their beneficial actions on the body [57]. In addition, cereal β-glucans could be used to promote in situ folate (i.e., Vit. B9) synthesis by certain yeasts and bacteria naturally present or added to the raw material [57,58]. The presence of β-glucans in meat emulsions meets the goal of increasing fiber levels in the diet at the same time as achieving better technological results. Unlike carrageenan and starch, which are hydrocolloids commonly used in meat products with strict legislative limits in quantities, β-glucans still require a legislative consensus on the maximum and minimum amounts that can be added to a given food matrix [25].

### 5.2. Use of β-Glucans in Solid Food Matrices: The Case of Bread

In solid food matrices, the addition of β-glucans has been studied mainly for cereal products (bread, rusks, pasta, biscuits, cake, etc.) In most European nations, bread is a staple diet and the main source of carbs. White bread made with wheat flour is the most popular starchy meal due to its exceptional organoleptic qualities as well as its taste. However, due to its porous structure and the large amount of gelatinized starch, it is categorized as a food with a high glycemic index (GI) [59]. Therefore, lowering the glycemic index of bread is of substantial scientific interest considering its great consumption. For this reason, the addition to bread of soluble dietary fiber, such as β-glucan concentrates from barley or oats, may be a successful strategy. In recent research, Binou et al. (2021) examined the post-prandial metabolic effects of two distinct kinds of functional breads enriched with β-glucans and resistant starch (RS) fractions, in the quantities recommended by the EFSA to have a health effect. Estimates of the test individuals’ subjective ratings of their hunger were taken together with measurements of glycemic and insulin levels, ghrelin, GLP-1, and PYY responses. Additionally, the sensory qualities of the enhanced goods were assessed. When compared to a glucose solution (GS), breads with G or RS had a smaller incremental glucose area under the curve (IAUC), and both kinds of bread had a low glycemic index (GI). The insulin, ghrelin, GLP-1, and PYY responses did not differ between the two significantly [60]. As reported by a Vitaglione et al. (2013) in a short-term study, the increase in the β-glucan fiber of snacks can modulate the appetite and energy intake of young people [61]. The research of Tessari and Lante (2017) focused on people with T2DM, evaluating the metabolic effects of a six-month replacement of ordinary white bread with a specially designed functional bread (Pane Salus^®^), with a modest content of starch but high fiber, respectively, 7 g/100 g, with a β-glucan/starch ratio of 7.6:100, *g*/*g*. The diabetic participants eating the functional bread showed significantly lower levels of fasting, postprandial, and average blood glucose concentrations, as well as a lower glycated hemoglobin (HbA1c) value than the control T2DM subjects [62]. Last but not least, the degree of the liking of the bread was satisfactory. In research by Liu et al. (2020), the technological properties of functional bread doughs formulated with 80% Hulled Barley Whole Grain Flour (HLB WGF) of waxy or normal lines and only 20% common wheat flour (WF) were described. Bread made with WF alone was considered as the control. The amount of water absorbed by each type of flour was detected using the Brabender farinograph. The WGF HLB presented a much higher water absorption rate than normal WF. The water absorption rate by waxy lines was significantly higher than that by normal lines. The development time, stability time, and the FQN (Farinograph Quality Number) of the mixtures obtained with HLB WGF were significantly lower than those obtained with simple WF, while their degree of softening was higher. The strength of the HLB WGF is lower than WF, which justifies its higher softening. In addition to a greater water absorption, the mixtures enriched with HLB WGF were more viscous (especially waxy lines), gelatinized more easily with better adhesive properties, and demonstrated a greater resistance to starch retrogradation than normal WF. Visually, the bread made with WF had a significantly bigger volume than the bread made with HLB WGF. Moreover, because waxy varieties of bread contain more β-glucans than normal ones, they have a lower specific volume. The breads enriched with HLB WGF did not significantly vary from regular breads made with WF in terms of the crust color. Ultimately the higher content of β-glucan and total dietary fiber could have a positive effect on the nutritional value of the resulting breads; however, they are also accompanied by a possible negative effect on the sensory quality and work performance of the product. Although due to their hydrocolloid nature, β-glucans determine a greater yield of the dough, they also cause a deterioration in the structural properties of the bakeries, including an increase in hardness, gumminess, and chewability, as well as a decrease in cohesion and elasticity. In any case, even if bread made with HLB WGF shows different sensory properties than bread made with WF alone, overall, the latter are acceptable to consumers. Therefore, these preparations could be substituted in a bakery to produce functional breads [63]. As reported by Messia et al. (2020) the enriched flour obtained by waxy barley could be used to produce functional foods such as pasta, bread, biscuits that follow the legal requirements of the FDA and EU Regulation [64].

## 6. Effects of Technological Treatments on the Structural Features and Functional Properties of β-Glucans

β-glucans may be subjected to a variety of stresses during processing and post-processing methods. These stresses can have an impact on β-glucans’ rheological and functional properties as well as some of their basic characteristics, including molecular structure, degree of polymerization, molecular interactions, functional and technological properties, physiological benefits, and sensory characteristics. Table 3 lists the primary impacts that certain techniques can have on dietary supplements of β-glucans [65].

### 6.1. Fermented Bakery and Doughs

The endogenous enzymes naturally present in wheat and other cereals, and consequently also in their flours, can greatly modify the structure of the β-glucan present. In the study by Andersson et al. (2004), it emerges that mixing and fermentation time greatly reduced the MWs of β-glucans in bread doughs made from sifted or whole barley flour [66]. Moreover, Andersson et al. (2008) studied endogenous rye flour β-glucanases in bread models enriched with oat bran, using rye flour with variable endogenous enzyme activity. Flour with the lowest endogenous enzyme activity gave breads with high MWs β-glucan, which indicates that the endogenous enzymes in the flour are responsible for breaking down β-glucan during baking. The study shows that the endogenous enzymes of the flour were also responsible for a greater solubility of β-glucan polymers through the breakdown of cell wall polysaccharides naturally bound with β-glucan molecules. For short fermentation times, rye breads enriched with oat bran high in β-glucans showed twice as much β-glucans in water as the rye loaves where oat bran was not added. This extractability is probably attributable to the solubilization of insoluble β-glucans following the activity of endogenous β-glucanases during the fermentation process. Despite a greater initial solubility for very long fermentation times, the content of β-glucans in water decreases progressively because the β-glucan molecules associate with each other or with other components, forming aggregates that are no longer soluble [67]. Furthermore, Tosh et al. (2008), in a study focused on preparing muffins with oat bran, reported the self-aggregation of β-glucans oligomers [38]. For this reason, the β-glucan structure, conformation, and a higher DP3/DP4 ratio may be more resistant to enzymatic hydrolysis [28].

### 6.2. Domestic Cooking

Cooking probably contributes to the slow release of β-glucans from the cell wall, increasing their solubility without drastic MW changes. Moreover, the type of recipe, the ingredient combinations, and the structure play a role in the solubility of β-glucans [68].

Based on these considerations, the health impact of β-glucans is also generated by the processes carried out from raw materials to food products [65].

### 6.3. Extrusion Firing

Extrusion is a high temperature, short time process that can have an impact on both the molecular weight of β-glucan and its solubility, depending on the parameters applied. The ingredients undergo many transitions of “order-disorder” (gelatinization of starch, denaturation of proteins, formation of complexes between lipids and amylose), thus altering the physical and chemical properties of extruded products [69]. High temperatures, high pressures, and mechanical forces applied during extrusion can break and recombine the covalent bonds between monomers, thus modifying the physical structures of macromolecules and leading to a change in their functional properties. Extrusion variables such as screw speed, screw configuration, cylinder temperature, and food humidity can influence the rheological and functional properties of ingredients, including viscosity, solubility, thermal and colloidal properties [70]. Zhang et al. (2011) reported a significant impact of the extrusion moisture and temperature reached by the food on the content of soluble β-glucans. The study showed that, compared to soluble fiber from untreated oat bran, soluble fiber from extruded oat bran manifests more aggregates, a higher gelatinization temperature, greater solubility, better swelling capacity and solvent retention, and an increase in apparent viscosity and consistency, as well as a decrease in fluidity index and greater foam stability. A not too intensive extrusion process can improve the functional and technological properties of soluble oat bran fiber [71]. In brief, extrusion cooking can be used to change the physicochemical and nutritionally relevant properties of β-glucans [72].

### 6.4. Freezing and Thawing Cycles

Subjecting cooked foods to several cycles of freezing and thawing can affect the solubility of β-glucans. Experimental findings have shown that frozen storage of oat muffins and bread significantly reduces the fraction of soluble β-glucan [68,73]. These changes of solubility are attributed to the change in molecular organization during storage, which leads to the repulsion of water molecules from the polysaccharide matrix [68]. Water crystallization reduces the hydration of polysaccharide macromolecules and leads to cryo-concentration of solubilized β-glucans during previous processing and cooking. These concentrated low-MW β-glucans will tend to aggregate via intermolecular hydrogen bonds that will reduce their solubility. The subsequent thawing process will allow greater mobility of the accumulated β-glucans, thus accelerating the formation of new intermolecular hydrogen bonds between the polymers ultimately leading to a more stable aggregation. In addition, the decrease in β-glucans solubility impacts the viscosity of the dough [74].

### 6.5. Final Consideration on Possible Technological Processes and Treatments

**Figure 2:**
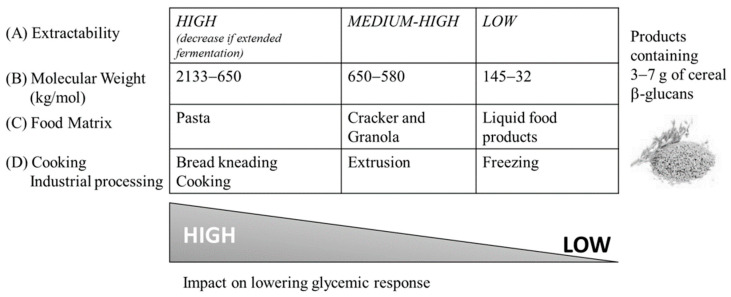
Impact of typical processing on the structure of cereal β-glucans. The capacity to lower glycemic response is indicated as a gradient from high (HIGH) to low (LOW). Adapted from [65].

As above reported, it is evident that the conformational changes of cereals β-glucans are highly process-dependent and are unpredictable. In addition, the type and the format of β-glucans (bran, flour, flakes) or the recipe also influences the structural change made by processing. Since cereals are usually never eaten raw, a cooking phase is generally necessary at least before their consumption. As summed up in Figure 2, the variations in solubility and MW determined by the different processing approaches in turn influence the viscous behavior of the food, as well as its palatability and the physiological effect that the latter can exert [65].

As shown in the figure, a high solubility of β-glucan has a greater effect on the reduction in glycemic response (A). Similarly, β-glucans with higher MW have a better ability to reduce blood sugar (B). A high decrease in glucose response seems possible for pasta added with 3–7 g of β-glucans, while crackers and granola have an intermediate result. Liquid food products, such as soups and beverages, have a low impact on reducing the glucose response (C). Baking and kneading processes maintain the overall ability of β-glucans to lower the glycemic response, while intensive extrusion and freezing processes adversely affect the hypoglycemic properties of β-glucans (D). However, the palatability of food products is always an open question because technological processes for improving palatability and lowering viscosity can affect the MW and solubility of β-glucans. For this reason, in the formulation and development of new functional products that are healthy but also attractive to consumers, the key physiochemical characteristics of β-glucans play an important role [65].

## 7. Conclusions

Fiber intake, and especially β-glucans, becomes an important dietary factor in T2DM control. However, to better manage and preserve the chemical-physical properties of β-glucans, it is necessary to pay close attention to the processes of extraction, processing, manipulation, and preservation of food, which subject this functional polymer to a series of stress factors. These conditions threaten its chemical and functional characteristics, including its ability to prevent diabetes.

Based on this information, the applied process and conservation parameters have to be re-evaluated to guarantee the maximum functionality of this molecule.

To extend the benefits of β-glucans to a wide range of consumers, it will also be necessary to consider critical aspects as the structural and sensory quality of the product, which play an important role in the purchase and consumption of foods enriched or fortified with this fiber.

## Figures and Tables

**Table 1:** Representative summary of the physico-chemical properties of β-glucans present in different food products adapted from [20].

Category	Common Name	Species	Name of the β-Glucan	Glycosidic Bonds	Solubility in Water
**Higher plants**	**Oats**	*Avena sativa*	**-**	β-1.3 and β-1.4 w/o branches	**Soluble**
**Higher plants**	**Barley**	*Hordeum* *Vulgar* and	**-**		**Soluble**
**Fungi**	**Maitake mushroom**	*Leafy griffon*	**Grifolan**	Main chain consists of β-1,3 glycosidic bonds with branches with single glucose units β-1,6	**Soluble**
**Fungi**	**Shiitake mushroom**	*Lentinula edodes*	**Lentinan**		**Soluble**
**Fungi**	**Schizophyllan**	*Schizophyllum commune*	**GSP**		**Soluble**
**Seaweed**	**Algae**	*Laminaria typed*	**Laminarin**	Main chain consisting of β-1,3 glycosidic bonds with β-1,6 side chains	**Soluble**
**Seaweed**	**Algae**	*Saccharina* *longicruris*	**Laminarin**		**Soluble**
**Seaweed**	**Cochayuyo**	*Durvillaea* *antarctica*	**Laminarin**		**Soluble**
**Yeasts**	**Brewer’s yeast**	*Saccharomyces cerevisiae*	**Zymosan**		**Insoluble**
**Microalgae**	**Euglena**	*Euglena* *gracilis*	**Paramylon**	Branchless chains β-1.3	**Insoluble**

**Table 2:** Characteristics of β-glucans from the most important cereal sources [18].

Cereal	Beta-Glucan Content (%)	Molar Mass (Kg/mol)	Molar Ratio DP3/DP4
Barley	5.0–11.0	400–2500	2.3–3.4
Oats	3.0–7.0	400–2500	1.5–2.3
Wheat	≅0.5	209–416	3.0–4.5
Rice	≅0.2	>1130	2.4–2.7

**Table 3:** Impact of typical processing on the characteristics of cereal β-glucans [65].

Type of Process	Effect
Bakery and Fermented Dough	Large molecular weight reduction
	Increased solubility
	Gelling
Extrusion Firing	Molecular weight reduction only under high temperature conditions
	Increased solubility
Freezing Cycles and Thawing	Decreased solubility due to aggregation into insoluble complexes

## Data Availability

Not applicable.

## Abbreviations

The following abbreviations are used in this manuscript:

ASE	Accelerated Solvent Extraction
BBG	Barley Beta-Glucan
BG	Beta-Glucan
DP	Degree of Polymerization
EFSA	European Food Safety Authority
FDA	Food and Drug Administration
FFAR2	Free Fatty Acid Receptor 2
FQN	Farinograph Quality Number
GI	Glycemic index
GLP-1	Glucagon-Like Peptide 1
GS	Glucose Solution
HbA1c	Hemoglobin A1c
HDL	High Density Lipoproteins
HLB WGF	Hulled Barley Whole Grain Flour
IAUC	Incremental Area under the Curve
IDF	Insoluble Dietary Fiber
IDF	International Diabetes Federation
LDL	Low Density Lipoproteins
MAE	Microwave-Assisted Extraction
MW	Molecular Weight
OBG	Oat Beta-Glucan
PBGR	Peak Blood Glucose Rise
PC	Phenolic Compounds
PHEB	barley flour hydrolyzed
PYY	Peptide YY
ROS	Reactive Oxygen Species
RS	Resistant Starch
SCFAs	Short Chain Fatty Acids
SDF	Soluble Dietary Fiber
SGLT2	Sodium/Glucose Co-Transporter 2
T2DM	Type 2 Diabetes Mellitus
UAE	Ultrasound-Assisted Extraction
WF	Wheat Flour
WHO	World Health Organization

## References

[1] Xie J., Wang M., Long Z., Ning H., Li J., Cao Y., Liao Y., Liu G., Wang F., Pan A. (2022). Global burden of type 2 diabetes in adolescents and young adults, 1990–2019: Systematic analysis of the Global Burden of Disease Study 2019. BMJ.

[2] WHO (2022). Diabetes.

[3] IDF (2023). IDF Diabetes Atlas|Tenth Edition. https://diabetesatlas.org/.

[4] Schulze M.B., Schulz M., Heidemann C., Schienkiewitz A., Hoffmann K., Boeing H. (2007). Fiber and magnesium intake and incidence of type 2 diabetes: A prospective study and meta-analysis. Arch. Intern. Med..

[5] Kuijsten A., Aune D., Schulze M.B., Norat T., van Woudenbergh G.J., Beulens J.W.J. (2015). Dietary fibre and incidence of type 2 diabetes in eight European countries: The EPIC-InterAct Study and a meta-analysis of prospective studies. Diabetologia.

[6] Jovanovski E., Khayyat R., Zurbau A., Komishon A., Mazhar N., Sievenpiper J.L., Mejia S.B., Ho H.V.T., Li D., Jenkins A.L. (2019). Shouldviscousfibersupplements be considered in diabetes control? Results from a Systematic Review and Meta-analysis of Randomized Controlled Trials. Diabetes Care.

[7] IDF (2023). Diabete di Tipo 2. https://idf.org/aboutdiabetes/type-2-diabetes.html.

[8] Abdul Rahim M.B.H., Chilloux J., Martinez-Gili L., Neves A.L., Myridakis A., Gooderham N., Dumas M.-E. (2019). Diet-induced metabolic changes of the human gut microbiome: Importance of short-chain fatty acids, methylamines and indoles. Acta Diabetol..

[9] El Khoury D., Cuda C., Luhovyy B.L., Anderson G.H. (2012). Beta glucan: Health benefits in obesity and metabolic syndrome. J. Nutr. Metab..

[10] Lattimer J.M., Haub M.D. (2010). Effects of Dietary Fiber and Its Components on Metabolic Health. Nutrients.

[11] Khorasaniha R., Olof H., Voisin A., Armstrong K., Wine E., Vasanthan T., Armstrong H. (2023). Diversity of fibers in common foods: Key to advancing dietary research. Food Hydrocoll..

[12] Sun L., Hu M., Zhao J., Lv L., Zhang Y., Liu Q., Zhang L., Yu C., Wang P., Li Q. (2021). Molecular Characteristics, Synthase, and Food Application of Cereal β-Glucan. J. Food Qual..

[13] FDA (1997). CFR-Code of Federal Regulations Title 21.

[14] EFSA (2011). Scientific Opinion on the substantiation of a health claim related to barley beta-glucans and lowering of blood cholesterol and reduced risk of (coronary) heart disease pursuant to Article 14 of Regulation (EC) No 1924/2006. EFSA J..

[15] EFSA (2009). Scientific Opinion on the substantiation of health claims related to beta glucans and maintenance of normal blood cholesterol concentrations (ID 754, 755, 757, 801, 1465, 2934) and maintenance or achievement of a normal body weight (ID 820, 823) pursuant. EFSA J..

[16] EFSA (2010). Scientific Opinion on the substantiation of a health claim related to oat beta glucan and lowering blood cholesterol and reduced risk of (coronary) heart disease pursuant to Article 14 of Regulation (EC) No 1924/2006. EFSA J..

[17] EFSA (2011). Scientific Opinion on the substantiation of health claims related to beta-glucans from oats and barley and maintenance of normal blood LDL-cholesterol concentrations (ID 1236, 1299), increase in satiety leading to a reduction in energy intake (ID 851, 852. EFSA J..

[18] Schmidt M. (2022). Cereal beta-glucans: An underutilized health endorsing food ingredient. Crit. Rev. Food Sci. Nutr..

[19] Ahmad A., Kaleem M. (2018). β-Glucan as a Food Ingredient. Biopolym. Food Des.

[20] Nakashima A., Yamada K., Iwata O., Sugimoto R., Atsuji K., Ogawa T., Ishibashi-Ohgo N., Suzuki K. (2018). β-Glucan in Foods and Its Physiological Functions. J. Nutr. Sci. Vitaminol..

[21] Izydorczyk M.S., Dexter J.E. (2008). Barley β-glucans and arabinoxylans: Molecular structure, physicochemical properties, and uses in food products—A Review. Food Res. Int..

[22] Sikora P., Tosh S.M., Brummer Y., Olsson O. (2013). Identification of high β-glucan oat lines and localization and chemical characterization of their seed kernel β-glucans. Food Chem..

[23] Gajdošová A., Petruláková Z., Havrlentová M., Červená V., Hozová B., Šturdík E., Kogan G. (2007). The content of water-soluble and water-insoluble β-d-glucans in selected oats and barley varieties. Carbohydr. Polym..

[24] Ryu J.-H., Lee S., You S., Shim J.-H., Yoo S.-H. (2012). Effects of barley and oat β-glucan structures on their rheological and thermal characteristics. Carbohydr. Polym..

[25] Mejía S.M.V., De Francisco A., Bohrer B. (2020). A comprehensive review on cereal β-glucan: Extraction, characterization, causes of degradation, and food application. Crit. Rev. Food Sci. Nutr..

[26] Lazaridou A., Biliaderis C.G. (2007). Molecular aspects of cereal β-glucan functionality: Physical properties, technological applications and physiological effects. J. Cereal Sci..

[27] Regand A., Chowdhury Z., Tosh S.M., Wolever T.M., Wood P. (2011). The molecular weight, solubility and viscosity of oat beta-glucan affect human glycemic response by modifying starch digestibility. Food Chem..

[28] De Arcangelis E., Djurle S., Andersson A.A., Marconi E., Messia M.C., Andersson R. (2019). Structure analysis of β-glucan in barley and effects of wheat β-glucanase. J. Cereal Sci..

[29] Benito-Román O., Alonso E., Lucas S. (2011). Optimization of the β-glucan extraction conditions from different waxy barley cultivars. J. Cereal Sci..

[30] Zhao Y., Zhou H.-M., Huang Z.-H., Zhao R.-Y. (2020). Different aggregation states of barley β-glucan molecules affects their solution behavior: A comparative analysis. Food Hydrocoll..

[31] Tosh S.M. (2013). The research legacy of Peter J. Wood. Bioact. Carbohydr. Diet. Fibre.

[32] Li L., Pan M., Pan S., Li W., Zhong Y., Hu J., Nie S. (2020). Effects of insoluble and soluble fibers isolated from barley on blood glucose, serum lipids, liver function and caecal short-chain fatty acids in type 2 diabetic and normal rats. Food Chem. Toxicol..

[33] Mikkelsen M.S., Jespersen B.M., Møller B.L., Lærke H.N., Larsen F.H., Engelsen S.B. (2010). Comparative spectroscopic and rheological studies on crude and purified soluble barley and oat β-glucan preparations. Food Res. Int..

[34] Zielke C., Stradner A., Nilsson L. (2018). Characterization of cereal β-glucan extracts: Conformation and structural aspects. Food Hydrocoll..

[35] Gamel T.H., Abdel-Aal E.-S.M., Ames N.P., Duss R., Tosh S.M. (2014). Enzymatic extraction of beta-glucan from oat bran cereals and oat crackers and optimization of viscosity measurement. J. Cereal Sci..

[36] Change G., Cimino M., York N., Alifah U., Mayssara A., Abo Hassanin Supervised A., Chinatown Y., Staff C., Change G. (2021). Guidelines on Nutrition Labelling CAC/GL 2—1985 as Last Amended 2021.

[37] Mäkelä N., Brinck O., Sontag-Strohm T. (2020). Viscosity of β-glucan from oat products at the intestinal phase of the gastrointestinal model. Food Hydrocoll..

[38] Tosh S.M., Brummer Y., Wolever T.M.S., Wood P.J. (2008). Glycemic Response to Oat Bran Muffins Treated to Vary Molecular Weight of β-Glucan. Cereal Chem..

[39] Rieder A., Knutsen S.H., Fernandez A.S., Ballance S. (2019). At a high dose even partially degraded beta-glucan with decreased solubility significantly reduced the glycaemic response to bread. Food Funct..

[40] Shoukat M., Sorrentino A. (2021). Cereal β-glucan: A promising prebiotic polysaccharide and its impact on the gut health. Int. J. Food Sci. Technol..

[41] Sun H., Saeedi P., Karuranga S., Pinkepank M., Ogurtsova K., Duncan B.B., Stein C., Basit A., Chan J.C., Mbanya J.C. (2021). IDF Diabetes Atlas: Global, regional and country-level diabetes prevalence estimates for 2021 and projections for 2045. Diabetes Res. Clin. Pract..

[42] Chakraborty S., Rajeswari V.D. (2022). Biomedical aspects of beta-glucan on glucose metabolism and its role on primary gene PIK3R1. J. Funct. Foods.

[43] DeFronzo R.A., Ferrannini E., Groop L., Henry R.R., Herman W.H., Holst J.J., Hu F.B., Kahn C.R., Raz I., Shulman G.I. (2015). Type 2 diabetes mellitus. Nat. Rev. Dis. Prim..

[44] Nauck M.A., Quast D.R., Wefers J., Pfeiffer A.F.H. (2021). The evolving story of incretins (GIP and GLP-1) in metabolic and cardiovascular disease: A pathophysiological update. Diabetes Obes. Metab..

[45] Richter M.M., Galsgaard K.D., Elmelund E., Knop F.K., Suppli M.P., Holst J.J., Winther-Sørensen M., Kjeldsen S.A.S., Wewer Albrechtsen N.J. (2022). The Liver–a-Cell Axis in Health and in Disease. Diabetes.

[46] Wolever T.M.S., Jenkins A.L., Prudence K., Johnson J., Duss R., Chu Y., Steinert R.E. (2018). Effect of adding oat bran to instant oatmeal on glycaemic response in humans—A study to establish the minimum effective dose of oat β-glucan. Food Funct..

[47] Rahmani J., Miri A., Černevičiūtė R., Thompson J., De Souza N.N.A., Sultana R., Varkaneh H.K., Mousavi S.M., Hekmatdoost A. (2019). Effects of cereal beta-glucan consumption on body weight, body mass index, waist circumference and total energy intake: A meta-analysis of randomized controlled trials. Complement. Ther. Med..

[48] Dong J., Cai F., Shen R., Liu Y. (2011). Hypoglycaemic effects and inhibitory effect on intestinal disaccharidases of oat beta-glucan in streptozotocin-induced diabetic mice. Food Chem..

[49] Brummer Y., Duss R., Wolever T.M.S., Tosh S.M. (2012). Glycemic Response to Extruded Oat Bran Cereals Processed to Vary in Molecular Weight. Cereal Chem..

[50] Mio K., Yamanaka C., Ichinose Y., Kohyama N., Yanagisawa T., Aoe S. (2020). Effects of barley β-glucan with various molecular weights partially hydrolyzed by endogenous β-glucanase on glucose tolerance and lipid metabolism in mice. Cereal Chem..

[51] Pino J.L., Mujica V., Arredondo M. (2021). Effect of dietary supplementation with oat β-glucan for 3 months in subjects with type 2 diabetes: A randomized, double-blind, controlled clinical trial. J. Funct. Foods.

[52] Jones B., Bloom S.R., Buenaventura T., Tomas A., Rutter G.A. (2018). Control of insulin secretion by GLP-1. Peptides.

[53] Burcelin R. (2016). When gut fermentation controls satiety: A PYY story. Mol. Metab..

[54] Brooks L., Viardot A., Tsakmaki A., Stolarczyk E., Howard J.K., Cani P.D., Everard A., Sleeth M.L., Psichas A., Anastasovskaj J. (2017). Fermentable carbohydrate stimulates FFAR2-dependent colonic PYY cell expansion to increase satiety. Mol. Metab..

[55] Depommier C., Everard A., Druart C., Plovier H., Van Hul M., Vieira-Silva S., Falony G., Raes J., Maiter D., Delzenne N.M. (2019). Supplementation with Akkermansia muciniphila in overweight and obese human volunteers: A proof-of-concept exploratory study. Nat. Med..

[56] Zhang H., Zhang N., Xiong Z.-Q., Wang G., Xia Y., Lai P., Ai L. (2018). Structural characterization and rheological properties of β-D-glucan from hull-less barley (*Hordeum vulgare* L. var. nudum Hook. f.). Phytochemistry.

[57] Karp S., Wyrwisz J., Kurek M.A. (2019). Comparative analysis of the physical properties of o/w emulsions stabilised by cereal β-glucan and other stabilisers. Int. J. Biol. Macromol..

[58] Kariluoto S., Edelmann M., Nyström L., Sontag-Strohm T., Salovaara H., Kivelä R., Herranen M., Korhola M., Piironen V. (2014). In situ enrichment of folate by microorganisms in beta-glucan rich oat and barley matrices. Int. J. Food Microbiol..

[59] Fardet A., Leenhardt F., Lioger D., Scalbert A., Rémésy C. (2006). Parameters controlling the glycaemic response to breads. Nutr. Res. Rev..

[60] Binou P., Yanni A.E., Stergiou A., Karavasilis K., Konstantopoulos P., Perrea D., Tentolouris N., Karathanos V.T. (2021). Enrichment of bread with beta-glucans or resistant starch induces similar glucose, insulin and appetite hormone responses in healthy adults. Eur. J. Nutr..

[61] Vitaglione P., Lumaga R.B., Montagnese C., Messia M.C., Marconi E., Scalfi L. (2013). Satiating effect of a barley beta-glucan-enriched snack. J. Am. Coll. Nutr..

[62] Tessari P., Lante A. (2017). A Multifunctional Bread Rich in Beta Glucans and Low in Starch Improves Metabolic Control in Type 2 Diabetes: A Controlled Trial. Nutrients.

[63] Liu J., Li Q., Zhai H., Zhang Y., Zeng X., Tang Y., Tashi N., Pan Z. (2020). Effects of the addition of waxy and normal hull-less barley flours on the farinograph and pasting properties of composite flours and on the nutritional value, textural qualities, and in vitro digestibility of resultant breads. J. Food Sci..

[64] Messia M.C., De Arcangelis E., Candigliota T., Trivisonno M.C., Marconi E. (2020). Production of ß-glucan enriched flour from waxy barley. J. Cereal Sci..

[65] Henrion M., Francey C., Lê K.-A., Lamothe L. (2019). Cereal B-Glucans: The Impact of Processing and How It Affects Physiological Responses. Nutrients.

[66] Andersson A.A.M., Armö E., Grangeon E., Fredriksson H., Andersson R., Åman P. (2004). Molecular weight and structure units of (1→3, 1→4)-β-glucans in dough and bread made from hull-less barley milling fractions. J. Cereal Sci..

[67] Andersson A., Rüegg N., Åman P. (2008). Molecular weight distribution and content of water-extractable β-glucan in rye crisp bread. J. Cereal Sci..

[68] Beer M.U., Wood P.J., Weisz J., Fillion N. (1997). Effect of Cooking and Storage on the Amount and Molecular Weight of (1→3)(1→4)-β-d-Glucan Extracted from Oat Products by an In Vitro Digestion System. Cereal Chem..

[69] Sharma P., Gujral H.S. (2013). Extrusion of Hulled Barley Affecting β-Glucan and Properties of Extrudates. Food Bioprocess Technol..

[70] Sayanjali S., Ying D., Sanguansri L., Buckow R., Augustin M.A., Gras S.L. (2017). The effect of extrusion on the functional properties of oat fibre. LWT.

[71] Zhang M., Bai X., Zhang Z. (2011). Extrusion process improves the functionality of soluble dietary fiber in oat bran. J. Cereal Sci..

[72] Roye C., Van Wayenbergh E., Henrion M., De Bondt Y., Chanvrier H., King R., Lamothe L.M., Courtin C.M. (2021). Extrusion-cooking affects oat bran physicochemical and nutrition-related properties and increases its β-glucan extractability. J. Cereal Sci..

[73] Gamel T.H., Badali K., Tosh S.M. (2013). Changes of β-glucan physicochemical characteristics in frozen and freeze dried oat bran bread and porridge. J. Cereal Sci..

[74] Tosh S.M., Brummer Y., Wood P.J., Wang Q., Weisz J. (2004). Evaluation of structure in the formation of gels by structurally diverse (1 → 3)(1 → 4)-β-D-glucans from four cereal and one lichen species. Carbohydr. Polym..

